# A Bony Mallet Thumb with Interposition of the Nail Plate

**DOI:** 10.1055/s-0040-1722181

**Published:** 2021-02-01

**Authors:** Ahmadreza Afshar, Ali Tabrizi, Hassan Taleb

**Affiliations:** 1 Department of Orthopedics, Imam Khomeini University Hospital, Urmia University of Medical Sciences, Urmia, Iran

**Keywords:** mallet finger, mallet thumb, bony mallet, complications

## Abstract

Thumb extensor injuries and bony avulsion in the distal phalanx of the thumb are rare compared with other fingers. The most reported complications are infection, nail deformity, joint incongruity, implant failure, recurrent flexion deformity, and residual pain. This report presents a case of 30-year-old man suffering from an injury in the left thumb distal phalanx with a displaced comminuted intra-articular fracture of the distal phalanx of the left thumb. The nail plate was interposed between the dorsal and palmar fragments. The interposition of the nail plate in the bony mallet thumb has not been described before. Surgical treatment and fixation with a 2-mm miniscrew resulted in successful treatment. Clinical suspicion of this complication is of great importance and can affect treatment outcomes.

Traumatic injuries to the extensor mechanism at the distal interphalangeal (DIP) joint can lead to a mallet finger with or without bony avulsion. 1 A mallet thumb refers to the avulsion of the extensor pollicis longus (EPL) tendon from its distal phalangeal insertion, which is rare in comparison with other fingers. 2

A mallet finger lesion is known as a variant of lesions such as skin wounds (open mallet) and/or fractures of the distal phalanx involving more than one-third of the articular surface or displaced fractures of the distal phalanx growth plate (Seymour lesions). 1 Also, distal phalanx fractures can occur due to flexor digitorum profundus (FDP) avulsion with concomitant and separated fracture of the distal phalanx dorsal base, but basically it is an uncommon injury. 2

A few reports have addressed thumb extensor injuries. Depending on the size of the fragments, conservative treatment may result in satisfactory outcomes in less than 50% of the articular joints without dislocation. But surgical treatments are recommended in patients with fragment or distal phalanx dislocations. 1 2 3 The common complications associated with conservative treatments and splint occur in approximately 45% of the cases. The frequent complications of surgical treatments are infection, nail deformity, joint incongruity, implant failure, nail deformity, recurrent flexion deformity, and residual pain. 1 3 Here, we present a case of a patient with interposition of the nail plate in the fracture site.

## Case Presentation

**Fig. 1:**
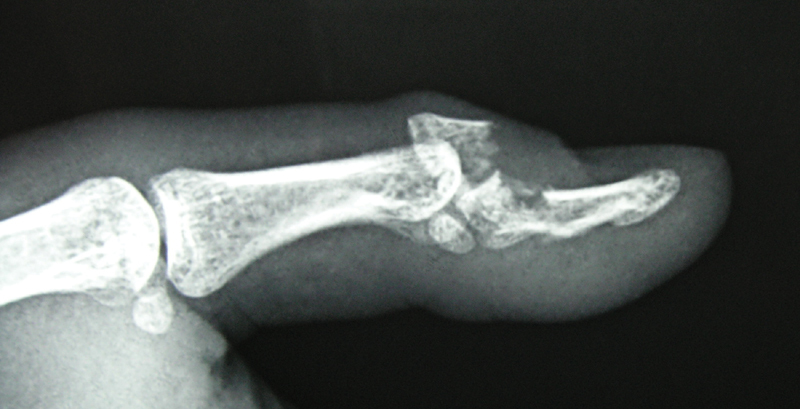
A displaced comminuted intra-articular fracture of the terminal phalanx of the left thumb. The dorsal fragment included more than 50% of the interphalangeal articular surface attached to the extensor pollicis longus tendon and is displaced dorsally producing a bony mallet thumb lesion.

A case of 30-year-old man suffering from an injury to his left thumb distal phalanx after falling during a soccer game is presented in this report. Upon physical examination, the left thumb was swollen and painful; moreover, he could not actively extend his interphalangeal (IP) joint. The initial radiographs demonstrated a displaced comminuted intra-articular fracture of the distal phalanx of the left thumb ( Fig. 1 ). The dorsal fragment with a size of 4 × 5 mm comprising more than 50% of the IP articular surface was displaced dorsally, producing a bony mallet thumb lesion.

**Fig. 2:**
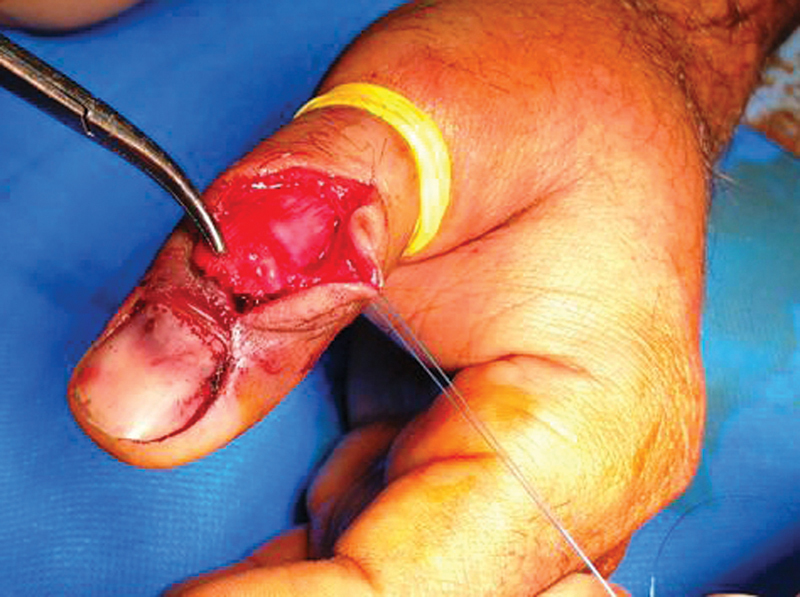
Under digital block and digital tourniquet and after the skin incision, there was a relatively large osseous fragment lying on the proximal part of the nail plate. The nail plate was interposed between the dorsal and palmar fragments.

**Fig. 3:**
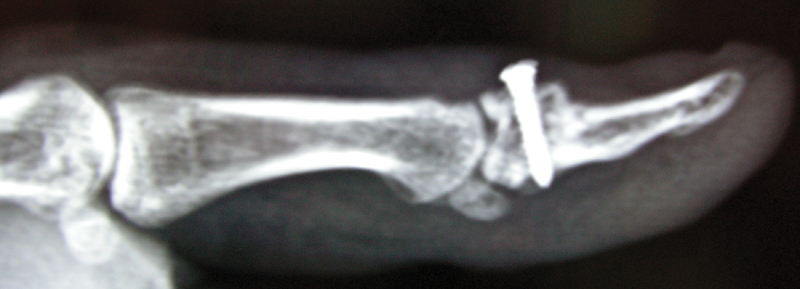
Six months postoperative lateral radiograph.

**Fig. 4:**
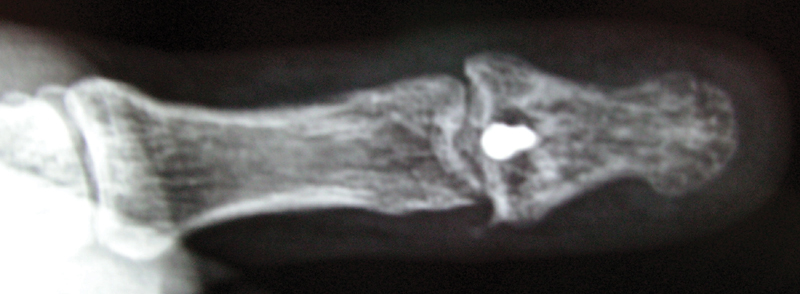
Six months postoperative anteroposterior radiograph.

**Fig. 5:**
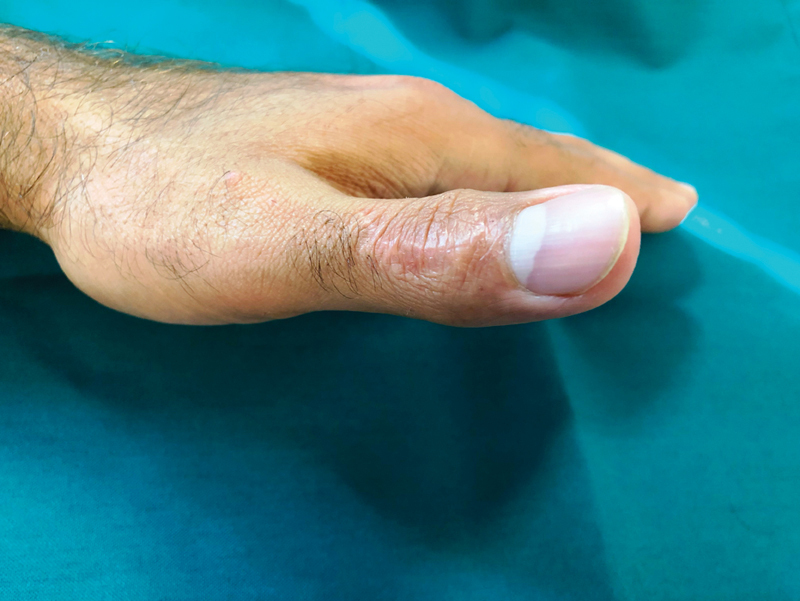
Eighteen months postoperative clinical photography demonstrating the active full extension of the thumb's interphalangeal joint without nail deformity.

**Fig. 6:**
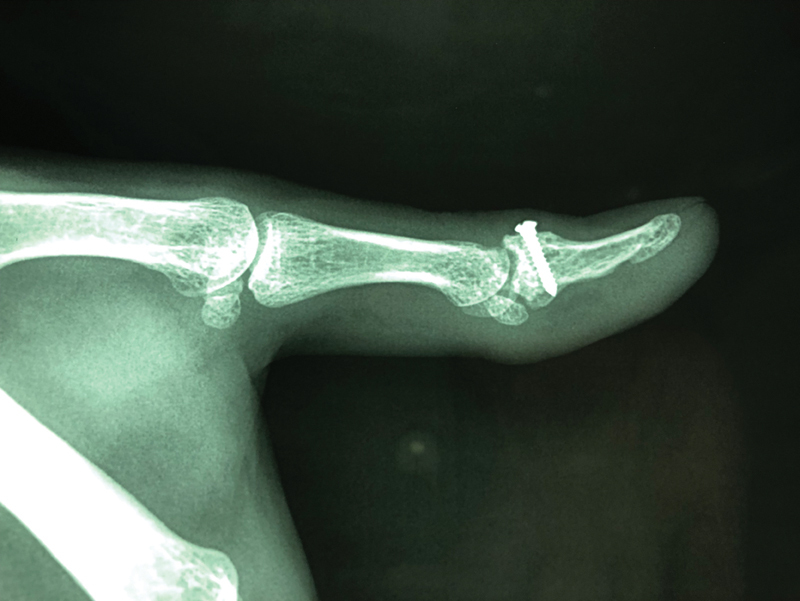
Eighteen months postoperative lateral radiograph demonstrating the full extension of the thumb's interphalangeal joint. There was no gap at the articular surface. All the bone fragments were consolidated.

Under digital block, the closed reduction and percutaneous pinning were initially attempted. However, intraoperative radiographs showed unsatisfactory reduction outcomes. Therefore, open reduction and internal fixation were implemented. At surgery, a relatively large dorsal osseous fragment attached to the EPL tendon lied on the proximal part of the nail plate, and the nail plate was interposed between the dorsal and palmar fragments ( Fig. 2 ). The fragment was fixed with a 2-mm miniscrew. The thumb's IP joint was immobilized with a spica cast for 6 weeks and then the range of motion related exercises were initiated ( Figs. 3 and 4 ). Eighteen months postsurgery, the IP joint was stable, and active range of motion was 0 extension to 45-degree flexion without extension lag with no nail deformity ( Fig. 5 ). The thumb radiographs demonstrated full extension of the IP joint with no gap at the articular surface, and all the bone fragments were consolidated ( Fig. 6 ). The patient was satisfied with the functional outcomes.

## Discussion

A bony mallet finger is a consequence of an avulsion of the extensor tendon from the distal phalanx with a bony fragment of the bone attached to the avulsed tendon. 4 The most common mechanism of injury in the mallet finger is a sudden flexion of the DIP joint with a resistance force directed along the long the finger axis. 4 This condition may occur in athletes especially baseball players. In our patient, the problem occurs due to direct trauma while playing soccer.

The readers may argue that our case is that of a comminuted fracture of the distal phalanx rather than a mallet injury; however, a mallet finger lesion can be considered a mirror lesion to an avulsion of FDP tendon. 1 We would like to compare our case with the Al-Qattan's type 5 avulsion of the insertion of the FDP tendon. 5 Al-Qattan reviewed FDP avulsions with a significant fracture of the distal phalanx and extended the classification. He introduced type 5 avulsion of the FDP tendon insertion described as FDP avulsion with a comminuted intra-articular fracture of the distal phalanx. 5 6

Some authors have extended the definition of mallet finger to fractures of the distal phalanx involving more than one or two-thirds of the articular surface or displaced fracture of the distal phalanx growth plate (Seymour lesion). 1

Regarding the Al-Qattan type 5 FDP avulsion with a comminuted intra-articular fracture of the distal phalanx, we suggested a mirror concept in our case. In our opinion, the disruption of the extensor mechanism through a significant bone lesion, where the extensor tendon is still attached to the dorsal fragment and the patient is unable to actively extend the thumb's IP joint, can be considered as a type of mallet lesion rather than a comminuted intra-articular distal phalanx fracture.

Compared with a mallet finger, a mallet thumb is an uncommon lesion since the thumb is shorter than the other fingers and its EPL is thicker than the terminal tendon of the extensor mechanism of the other fingers. 4 7 8 The bony mallet thumb is an even rarer lesion. 3 A bony mallet finger refers to an avulsion of the extensor tendon from the distal phalanx with a fragment of bone attached to the avulsed tendon. 1 The most common mechanism of a bony mallet finger is an axial force followed by sudden extreme hyperextension of the DIP joint. This condition occurs in sports injuries, in particular among baseball players. 1

Treatments of bony mallet thumbs may vary from splinting to surgical treatment. 1 When the fragment involves more than 30 to 50% of the articular surface of the IP joint, it is unstable and requires surgical fixation to prevent the joint subluxation. Insufficient treatment may lead to extensor lag, early osteoarthritic changes of the DIP joint, or even a swan-neck deformity. 9

Only a few case reports and sporadic cases of bony mallet thumb have been described among the large case series describing the mallet fingers or mallet thumbs. The bony mallet thumbs can be surgically treated with tension band technique, pull-in suture, extension block pinning with one or two Kirschner wires, hook plate, screw, external fixation, compression pins, and biodegradable device. 9 In a recent study by Vester et al, treatment by hook plate technique was reported as a reliable and safe method to treat bony avulsion fractures of the distal phalanx, with high patient satisfaction. 9 But the esthetic aspects and the nail deformities are more challenging. 9 In our presented patient, however, no nail deformity occurred with the screw, which seemed to be due to the large size of the bony fragment with stable fixation and lack of damage to the germinal matrix.

Although these various techniques resulted in excellent functional outcomes, the authors' successful experiences were limited to a few cases of bony mallet thumb. 3 4 6 7 8 The complication rate of surgical treatment has been reported up to 53%. The probable serious complications are hardware-associated infections, osteoarthritis, stiffness, nail dystrophy, and skin necrosis. 1

## Conclusion

This case report described a bony mallet thumb lesion with interposition of the nail plate between the dorsal and volar fragments. The avulsed fragment was large enough to receive a 2-mm miniscrew. To the best of our knowledge, the interposition of the nail plate in the bony mallet thumb has not been described before.
